# Adoptive cellular immunotherapy combined with chemotherapy versus chemotherapy alone in Chinese patients with metastatic colorectal cancer: a cost-effectiveness analysis to inform drug pricing

**DOI:** 10.3389/fonc.2025.1590319

**Published:** 2025-06-09

**Authors:** Liman Huo, Ping Liang, Yangyang Duan, Yanmei Xu, Jianhua Tang, Qi Lv, Rui Feng

**Affiliations:** ^1^ Department of Pharmacy, The Fourth Hospital of Hebei Medical University, Shijiazhuang, China; ^2^ School of Pharmacy, Hebei Medical University, Shijiazhuang, China; ^3^ Department of Quality Control, Hebei Institute for Drug and Medical Device Control, Shijiazhuang, China; ^4^ Department of Pharmacy, The First Affiliated Hospital of Hebei North University, Zhangjiakou, Hebei, China; ^5^ School of Disaster and Emergency Medicine, Tianjin University, Tianjin, China

**Keywords:** adoptive cellular immunotherapy, metastatic colorectal cancer, cost-effectiveness analysis, Markov model, drug pricing, incremental cost-effectiveness ratio

## Abstract

**Objective:**

To evaluate the cost-effectiveness of adoptive cellular immunotherapy (ACI) combined with chemotherapy versus chemotherapy alone in Chinese patients with metastatic colorectal cancer (mCRC) and provide evidence-based support for drug pricing strategies.

**Methods:**

A Markov model was constructed using data from the NCT03950154 phase III clinical trial, which randomized 202 patients into two groups: ACI combined with oxaliplatin/capecitabine/bevacizumab (n=100) and chemotherapy alone (n=102). Clinical outcomes, including progression-free survival (PFS), overall survival (OS), and adverse events, were analyzed. Costs, quality-adjusted life-year (QALY), and incremental cost-effectiveness ratio (ICER) were calculated from the perspective of the Chinese healthcare system. Probabilistic sensitivity analysis was employed to assess model stability, accompanied by scenario analysis, with price simulations conducted under three willingness-to-pay (WTP) thresholds (1.5×, 1.94×, and 3× China’s per capita gross domestic product [GDP]).

**Results:**

The ACI group demonstrated superior clinical outcomes compared to chemotherapy alone, with a median PFS of 14.8 vs. 9.9 months (hazard ratio [HR]=0.60, p=0.009) and a median OS not reached vs. 25.6 months (HR=0.57, p=0.043). Over a 20-year simulation, the ACI group provided an additional 1.72 QALY, yielding an ICER of $35,881.71/QALY. At the base-case price ($6,819.45 per cycle), ACI remained cost-effective within China’s WTP threshold ($36,721.86/QALY). Scenario analysis revealed that extending the simulation time horizon to 10 and 15 years reduced the ICER to $40,804.77/QALY and $37,770.23/QALY, respectively. Systematic cross-validation of 2,448 model combinations (72 control group/34 ACI group survival curves) indicated that 71.84% of scenarios met predefined cost-effectiveness criteria (ICER range: $22,204 – $58,360/QALY). Price sensitivity analysis further demonstrated that cost-effectiveness advantages persisted when ACI cycle costs were reduced to $1,670.33 (corresponding to WTP=1.5×GDP=$18,360.93), $3,271.06 (corresponding to WTP = 1.94×GDP = $23,746.80), and $7,098.9 (corresponding to WTP = 3×GDP = $36,721.86).

**Conclusion:**

At current pricing, ACI combined with chemotherapy provides significant clinical and economic benefits for Chinese mCRC patients, with cost-effectiveness validated through multidimensional scenario analyses and model evaluations. Further evidence is required to validate and refine the findings.

## 1 Introduction

According to the 2020 Global Cancer Statistics, colorectal cancer (CRC) accounted for over 1.9 million new cases and 930,000 deaths, making it the third most common type of cancer and the second leading cause of cancer-related mortality (1). Due to the lack of definitive clinical symptoms and signs in the early stages, approximately 20% of newly diagnosed patients with CRC present with distant metastases (2).

Fluoropyrimidine-based combination chemotherapy plus targeted therapy is currently recommended as the initial treatment for metastatic CRC (mCRC) (3). Among various chemotherapy regimens, XELOX (capecitabine plus oxaliplatin) with or without bevacizumab is one of the first-line options for mCRC. However, the clinical benefits of XELOX plus bevacizumab remain limited. In recent years, immune checkpoint blockade (ICB) therapy has revolutionized the treatment landscape for many solid tumors due to its remarkable efficacy. ICB is strongly recommended for mCRC patients with DNA mismatch repair deficiency/microsatellite instability. In contrast, for patients with mismatch repair-proficient mCRC, neither single-agent ICB therapy nor ICB combined with first-line chemotherapy plus bevacizumab has demonstrated significant progression-free survival (PFS) benefits (4–6). Adoptive cellular immunotherapy (ACI), which involves the administration of immunologically active cells, offers a potential alternative for mCRC treatment. Several types of immunologically active cells have been extensively studied in CRC. A recent meta-analysis of 70 patients from 6,743 studies supports this approach, showing that adoptive cytokine-induced killer/dendritic cell-cytokine-induced killer cell immunotherapy combined with standard regimens (particularly chemotherapy) provides significant clinical benefits for patients with CRC (7, 8). According to a phase III clinical trial (ClinicalTrials.gov: NCT03950154) (9), programmed cell death 1 (PD1) blockade-activated dendritic cell-cytokine-induced killer (PD1-T) cells combined with XELOX plus bevacizumab as a first-line regimen significantly improved PFS and overall survival (OS) in patients with mCRC, with favorable tolerability.

The trial results demonstrated that the median PFS in the immunotherapy group was 14.8 months (95% confidence interval [CI]: 11.6–18.0), significantly superior to the 9.9 months (95% CI, 8.0–11.8) observed in the control group (hazard ratio [HR]: 0.60; 95% CI: 0.40–0.88; p=0.009). Additionally, the median OS in the immunotherapy group was not reached, whereas it was 25.6 months (95% CI: 18.3–32.8) in the control group (HR: 0.57; 95% CI: 0.33–0.98; p=0.043). Regarding safety, the incidence of grade ≥3 adverse events (AEs) was 20.0% in the immunotherapy group versus 23.5% in the control group, with no toxicity-related deaths reported.

These findings indicate that PD1-T cell immunotherapy combined with chemotherapy provides significant improvements in PFS and OS with manageable safety. In the pharmacoeconomic analysis, these clinical efficacy data will serve as critical inputs for evaluating the cost-effectiveness of this combination regimen. By calculating the incremental cost-effectiveness ratio (ICER) of immunotherapy plus chemotherapy versus chemotherapy alone, this analysis will offer scientific evidence to inform pricing strategies and healthcare reimbursement decisions, thereby optimizing the allocation of medical resources and enhancing patient quality of life and survival benefits.

## 2 Methods

### 2.1 Study overview

This study strictly adhered to the Consolidated Health Economic Evaluation Reporting Standards (CHEERS) guidelines and focused on Chinese adult patients (age≥18 years) with previously untreated mCRC (10). The patient characteristics were assumed to align with those enrolled in the NCT03950154 clinical trial (**Supplementary Table S1**).

A total of 202 patients were included and randomized in a 1:1 ratio to either the ACI group (n=100) or the control group (n=102). The ACI group received bevacizumab (7.5 mg/kg) intravenously on day 1, oxaliplatin (130 mg/m²) intravenously on day 1, capecitabine (1,000 mg/m²) orally on days 1–14, and PD1-T cells 1×10^10^ intravenously on day 17, repeated every 21 days for six cycles. Maintenance therapy (administered every 21 days) consisted of bevacizumab (7.5 mg/kg) intravenously on day 1 and capecitabine (1,000 mg/m²) orally on days 1–14. The control group received an identical regimen, excluding PD1-T cells. AE incidence data were derived from the NCT03950154 trial.

Based on the Report on Nutrition and Chronic Diseases of Chinese Residents (2020) (11), the average patient height and weight were set at 165 cm and 65 kg, respectively, yielding an average body surface area of 1.79 m² for drug dosage and cost calculations in the control group.

All patients continued treatment until disease progression or the occurrence of intolerable adverse events (AEs). Post-progression, subsequent therapies were administered to 56.0% and 64.7% of patients in the ACI group and control group, respectively. These subsequent regimens, aligned with the NCT03950154 trial, included treatments accounting for ≥2% of cases and endorsed by the National Comprehensive Cancer Network and Chinese Society of Clinical Oncology guidelines (**Supplementary Table S6** **) (** 12, 13).

### 2.2 Model construction

**Figure 1:**
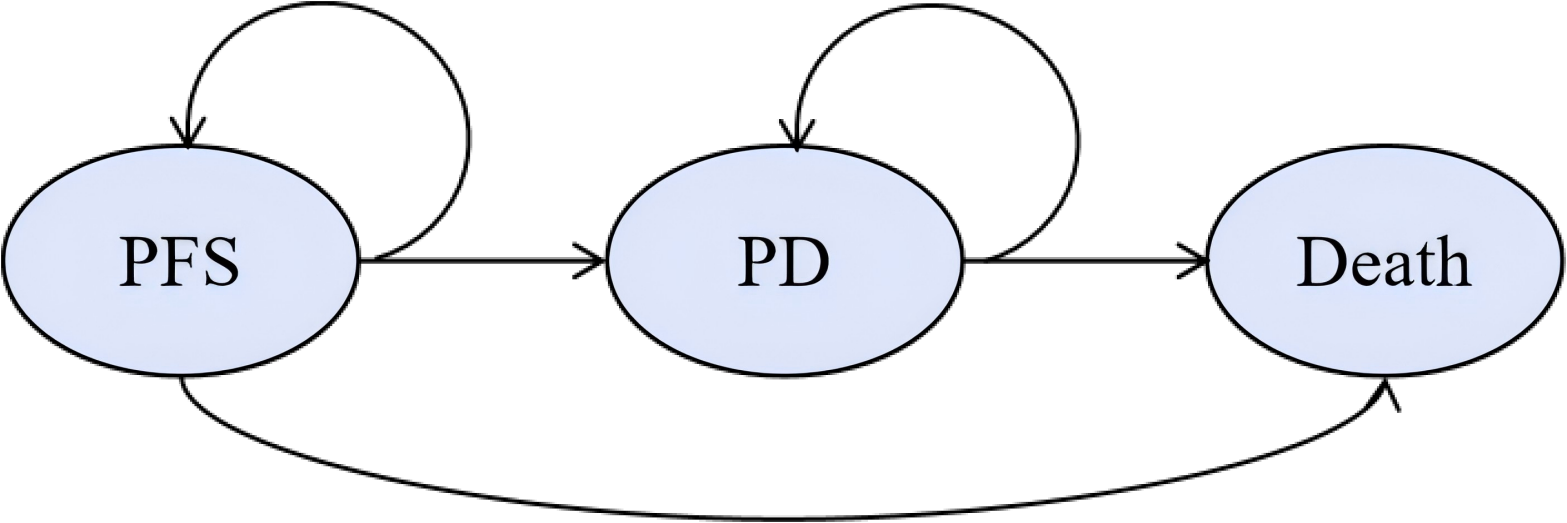
Markov model structure. The ellipses represent health states and the arrows represent the direction of movement between health states. PD, progressive disease; PFS, progression-free survival.

We developed a Markov model to compare healthcare costs and clinical outcomes between PD-1 blocked-activated DC-CIK cells combined with bevacizumab and chemotherapy versus bevacizumab plus chemotherapy alone as first-line treatment for patients with mCRC (14). The model included three mutually exclusive health states: PFS, PD, and death (**Figure 1**). Patients entered the model in the PFS state, transitioning between health states based on disease progression or death, with each cycle allowing only one health state occupancy and corresponding treatment. Based on the expert advice of clinicians and in alignment with the relevant requirements outlined in the Guidelines for Pharmacoeconomic Evaluation in China (2020), the study duration was determined to reasonably reflect the natural progression of the disease (15). In accordance with the clinical drug administration regimen, the model cycle was set to 21 days, with a total of 374 cycles simulated. Upon termination of the model, over 95% of the patients had transitioned to the death state. Consequently, the simulation duration of the model was established as 20 years to ensure comprehensive coverage of the disease’s long-term outcomes.

The analysis adopted the perspective of the Chinese healthcare system. Primary outputs included total costs, quality-adjusted life-year (QALY), and incremental cost-effectiveness ratio (ICER). An annual discount rate of 5% (range: 0–8%) was applied to both costs and outcomes (15). China’s willingness-to-pay (WTP) threshold was set at $36,721.86/QALY corresponding to three times the national gross domestic product per capita gross domestic product (GDP) in 2024 (15, 16). The model was constructed using R 4.1.2 (https://www.r-project.org/), leveraging the “flex surv” and “IPD from KM” packages to reconstruct individual patient data (IPD) and extrapolate survival outcomes.

### 2.3 Effectiveness

Using Guyot’s methodology (17), Kaplan-Meier (K-M) curves for OS and PFS from the NCT03950154 trial were digitized with Web Plot Digitizer (https://wpd.starrydata2.org/) to reconstruct IPD estimates. Virtual IPD included event and censoring times, closely replicating the original –M curves (**Supplementary Figure S1**). Reconstructed IPD were fitted to multiple survival distributions (exponential, Weibull, Gompertz, gamma, log-logistic, log-normal, generalized gamma, fractional polynomial, restricted cubic spline, and Royston–Parmar spline models). Model selection was guided by the Akaike Information Criterion (AIC), Bayesian Information Criterion (BIC), and visual inspection. Models with lower AIC and BIC values, coupled with visually reasonable fits, were considered to demonstrate superior performance. We systematically assessed the goodness-of-fit for multiple candidate models and identified the optimal model to extrapolate the K-M curves beyond the follow-up duration of the NCT03950154 trial. The final model selections and their corresponding performance metrics are detailed in the supplementary tables and figures (**Supplementary Tables S2**-**S4**, **Supplementary Figures S2**, **S3**).

### 2.4 Costs and utilities

Only direct medical costs were considered, including drug acquisition, follow-up procedures, adverse event (AE) management, best supportive care (BSC), and end-of-life care. Drug prices were sourced from public databases (e.g., WUXU) (18). Severe AEs (grade ≥3) with incidence >2% (e.g., anemia, neutropenia, leukopenia) were included, with costs and durations derived from published studies (19–23).

Utility values for health states and AE-related disutilities were extracted from prior studies (21–28), with AE disutilities assumed to occur in the first cycle and subtracted from baseline utilities after duration adjustment. Detailed cost and utility parameters are provided in **Supplementary Table S5**.

### 2.5 Sensitivity analysis

In the one-way sensitivity analysis (OWSA), key parameters were varied within their upper and lower bounds to evaluate the robustness of the model. Parameters with higher uncertainty were assigned a variation range of ±30% around their baseline values, whereas the remaining parameters were assigned a variation range of ±25%. The results were visualized using a tornado diagram. Additionally, a probabilistic sensitivity analysis (PSA) was conducted with 1,000 Monte Carlo iterations, presented in the form of cost-effectiveness acceptability curves (CEACs) and scatter plots. In this analysis, costs were modeled using a gamma distribution, while probabilities, proportions, and utilities were modeled using a beta distribution.

### 2.6 Price simulation

In the absence of prior pricing data for ACI in mCRC, a base-case price of $6,819.45 was assumed (29). In accordance with Chinese pharmacoeconomic guidelines (15), the WTP threshold was set at three times the 2024 per capita GDP. Based on Cai etal.’s (30) statistical life value framework, the monetary value of a QALY was estimated at 1.5 times the per capita GDP, based on data from the general population in China, WTP threshold for end-stage diseases was 1.94 times the per capita GDP (31), leading to extended threshold analyses at 1.5× GDP and 1.94× GDP of the baseline WTP. To assess the impact of immunotherapy pricing on the ICER, the model was run iteratively with adjustments to the immunotherapy (In this study, immunotherapy price refers specifically to adoptive cellular immunotherapy price) price until the ICER converged with the WTP threshold. The price defined at this point, where ICER equals WTP, was considered the upper limit of the immunotherapy price.

### 2.7 Scenario analysis

Scenario 1: The model duration was set to 10 and 15 years to evaluate the impact of time variation on outcomes.

Scenario 2: A sensitivity analysis of distribution models was performed, assessing 7 to 15 candidate models while excluding abnormal survival curves according to clinical plausibility. Cross-validation was conducted by employing 72 combinations of control group and 34 combinations of immunotherapy group, ensuring that OS curves were greater than or equal to PFS curves. Subsequently, the research team applied an algorithm to randomly pair all combinations of control group and immunotherapy group models, and the resulting transition probability matrices were input into a Markov model to calculate the ICER.

## 3 Results

### 3.1 Base-case analysis results

The results of the base-case analysis are summarized in **Table 1**. Over a 20-year time horizon, the ACI group achieved 3.91QALYs, compared with 2.18 QALY in the control group, yielding an incremental gain of 1.73 QALY with ACI.

**Table 1:** Results of the cost-effectiveness analysis.

Group	Total cost ($)	Total QALY	Incremental cost ($)	Incremental QALY	ICER ($/QALY)
Immunotherapy group	108,136.35	3.91	62,029.78	1.73	35,881.71
Control group	46,106.57	2.18			

Control group, XELOX plus bevacizumab; Immunotherapy group, XELOX plus bevacizumab and PD-1 blocked-activated DC-CIK cells; ICER, incremental cost-effectiveness ratio; QALY, quality-adjusted life year.

When priced at $6,819.45 per cycle, the total cost for the ACI group and control group was $108,136.35 and $46,106.57, respectively, resulting in an ICER of $35,881.71/QALY. At a WTP threshold of $36,721.86/QALY, ACI group demonstrated cost-effectiveness.

### 3.2 Sensitivity analysis

**Figure 2:**
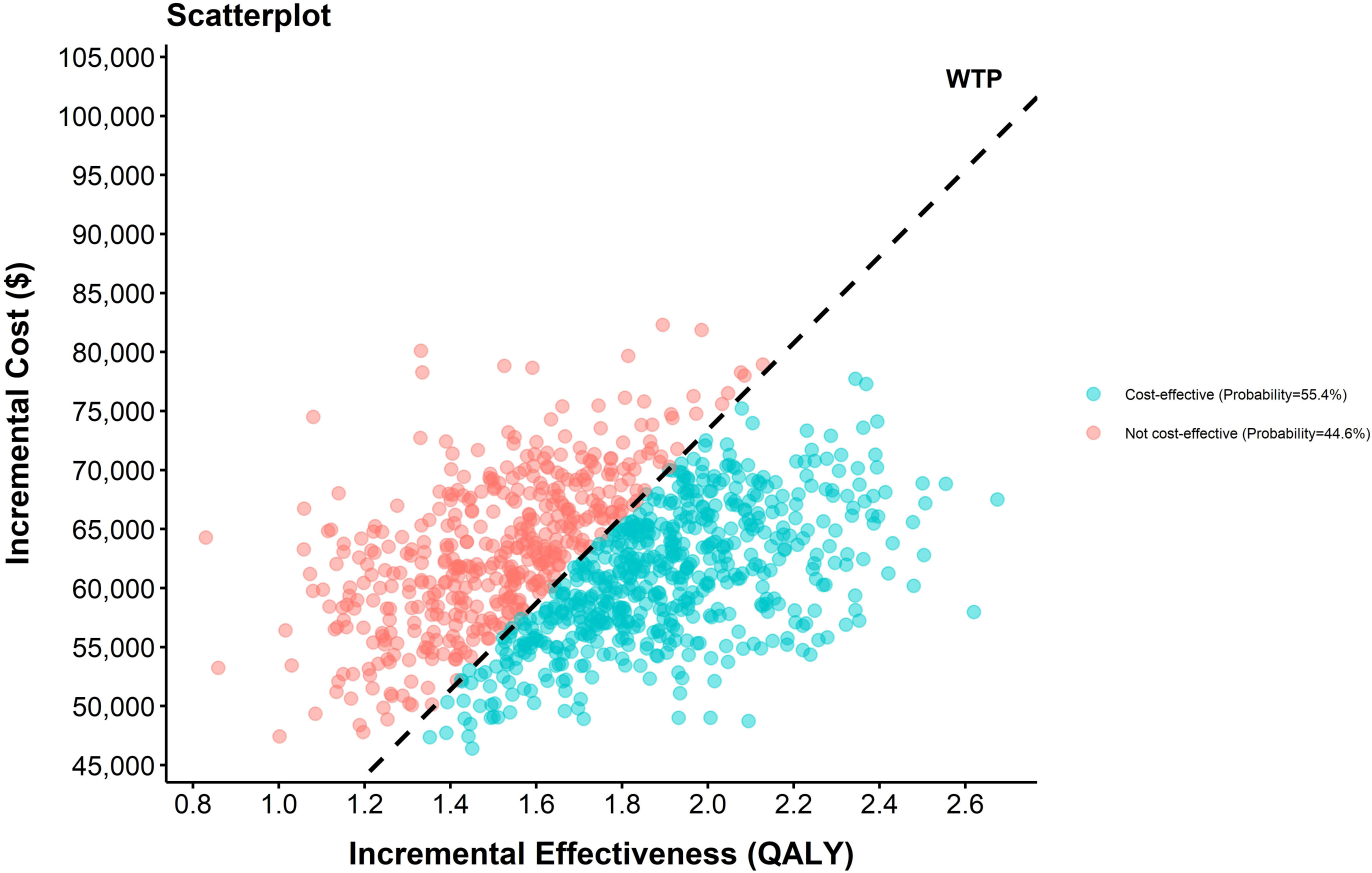
Cost-effectiveness scatter plot.

**Figure 3:**
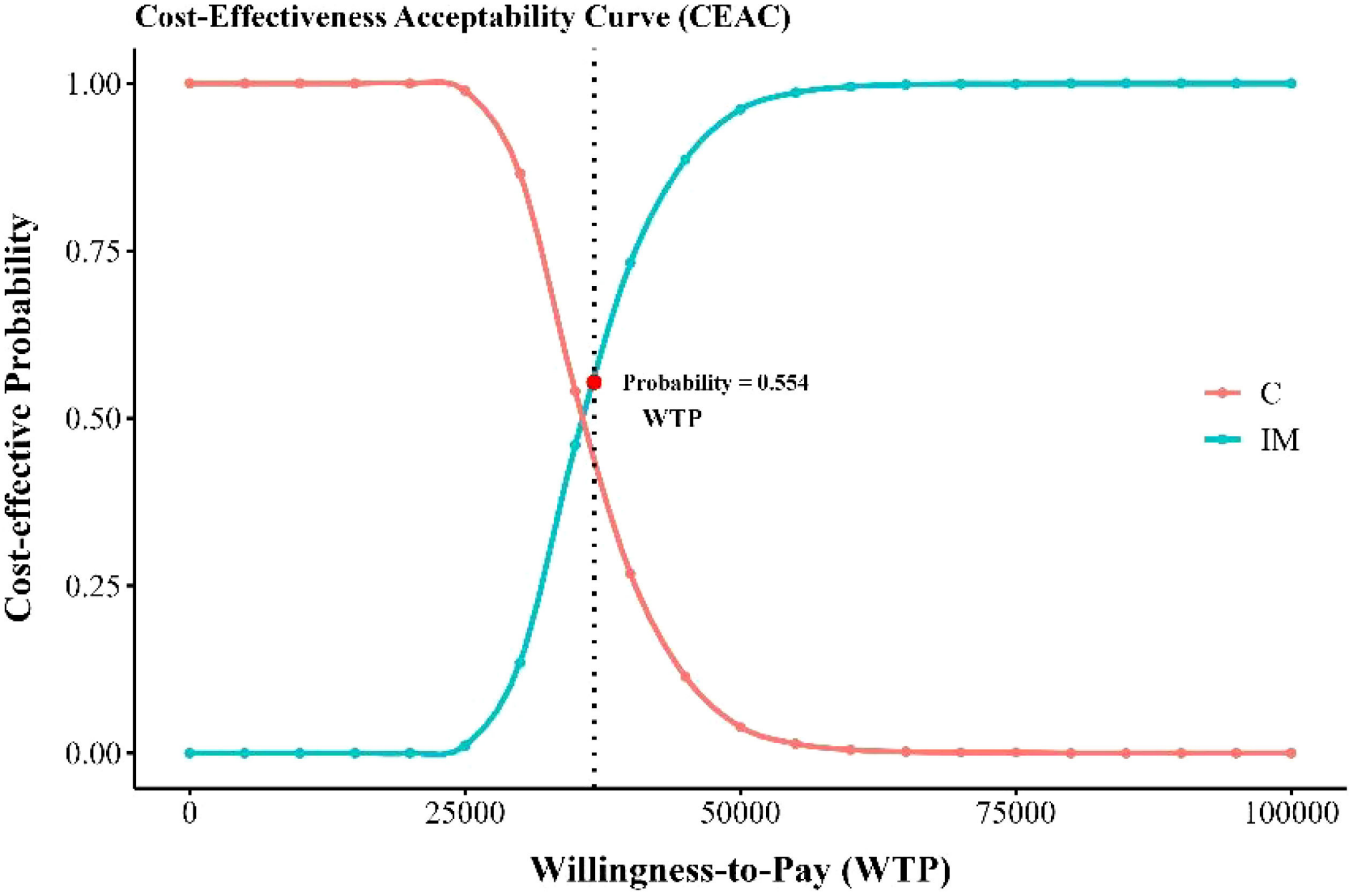
Cost-effectiveness acceptable curve. IM, immunotherapy group, XELOX plus bevacizumab and PD-1 blocked-activated DC-CIK cells; C, Control group, XELOX plus bevacizumab.

The sensitivity analysis results demonstrated the cost-effectiveness outcomes of the ACI group, as shown in the scatter plot (**Figure 2**) and the acceptability curve (**Figure 3**). At a WTP threshold of $36,721.86/QALY, the ACI group became marginally cost-effective when the treatment cost of PD-1 blocked-activated DC-CIK cells was $5,897.707, yielding an ICER of $35,881.7/QALY. Under this scenario, the probability of cost-effectiveness acceptability reached 55.4%.

**Figure 4:**
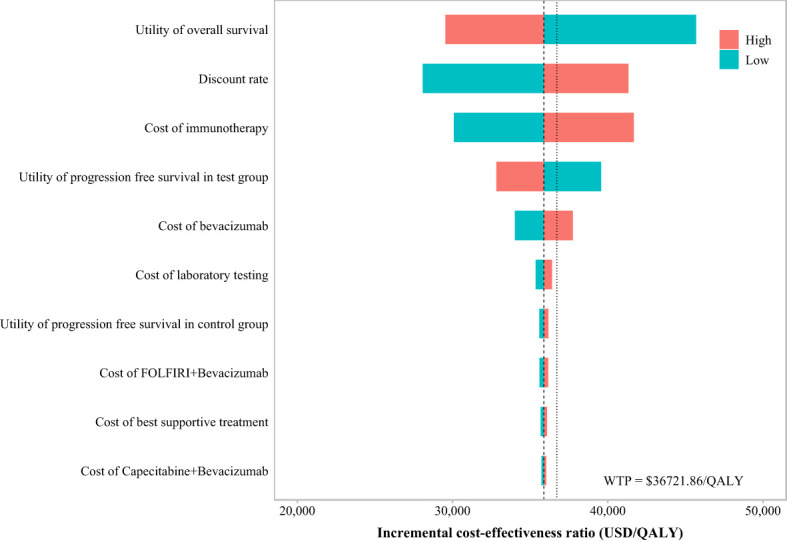
Results of OWSA in the tornado diagrams.

The OWSA (**Figure 4**) revealed that when the ICER result was $35,881.7/QALY—just below the WTP threshold of $36,721.86/QALY—the key parameters influencing the results, ranked by their impact magnitude, were as follows: the utility value of PD, the discount rate, the cost of immunotherapy (immunotherapy refers specifically to ACI), the utility value of PFS in ACI group, and the cost of bevacizumab. Changes in these parameters significantly affected the model’s economic outcomes.

### 3.3 Price simulation

**Figure 5:**
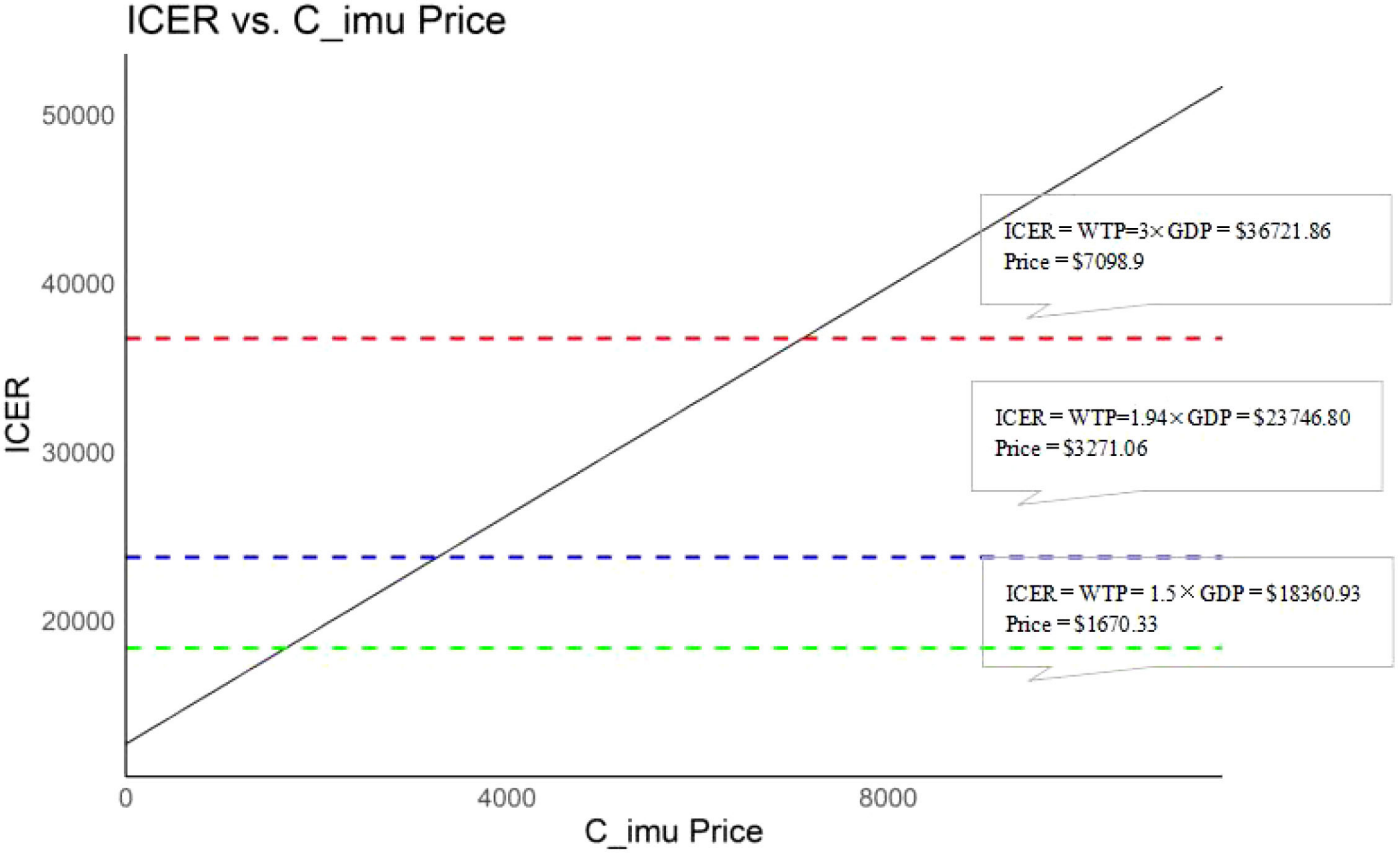
Results of price simulation.

When considering the impact of immunotherapy pricing on cost-effectiveness outcomes alone, a positive correlation was observed between the treatment cost of PD-1 blocked-activated DC-CIK cells (range: $0–$7,098.90) and the ICER. At a WTP threshold of $36,721.86, the intervention remained cost-effective when the immunotherapy cost was below $7,098.90. Further analysis revealed that the immunotherapy group retained cost-effectiveness when costs fell below $1,670.33 (1.5× GDP, WTP = $18,360.93) and $3,271.06 (1.94× GDP, WTP = $23,746.80) (**Figure 5**).

### 3.4 Scenario analysis

Scenario Analysis 1: When the time horizon of the model was adjusted to 10 years and 15 years, the ICERs of the ACI group compared to the control group were $40,804.77/QALY and $37,770.23/QALY, respectively. As the time horizon increased, the ICER decreased gradually (**Table 2**).

**Table 2:** Cost-effectiveness analysis results under different model runtime scenarios.

Scenarios	Total cost ($)		QALY		ICER ($/QALY)
	Immunotherapy group	Control group	Immunotherapy group	Control group	
Model runtime (year) = 10	104076.71	44725.60	3.49	2.03	40804.77
Model runtime (year) = 15	106829.76	45900.13	3.78	2.16	37770.23

Control group, XELOX plus bevacizumab; Immunotherapy group, XELOX plus bevacizumab and PD-1 blocked-activated DC-CIK cells; ICER, incremental cost-effectiveness ratio; QALY, quality-adjusted life year.

**Figure 6:**
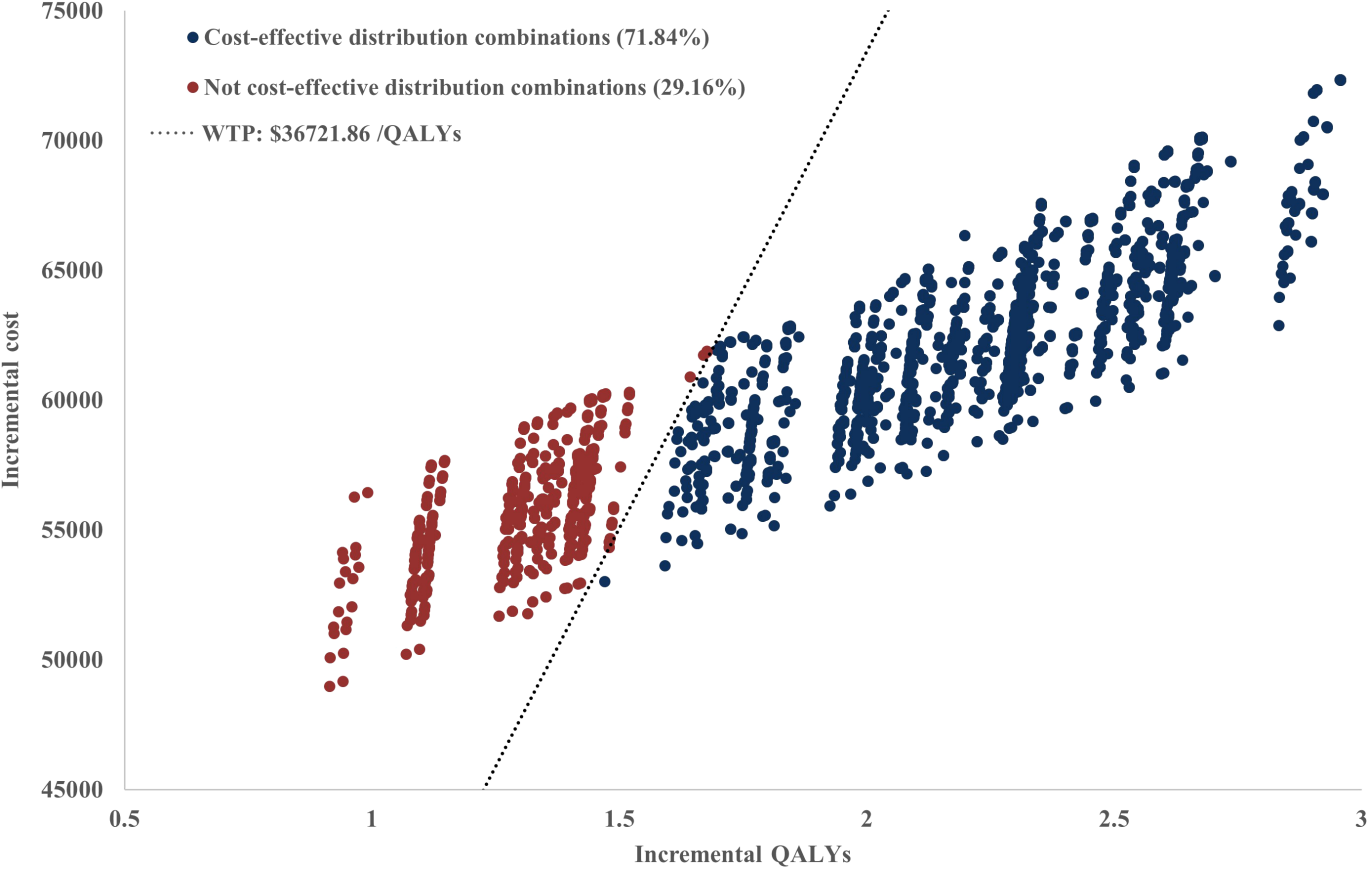
The scatter plot of the sensitivity analysis for the distribution model.

**Figure 7:**
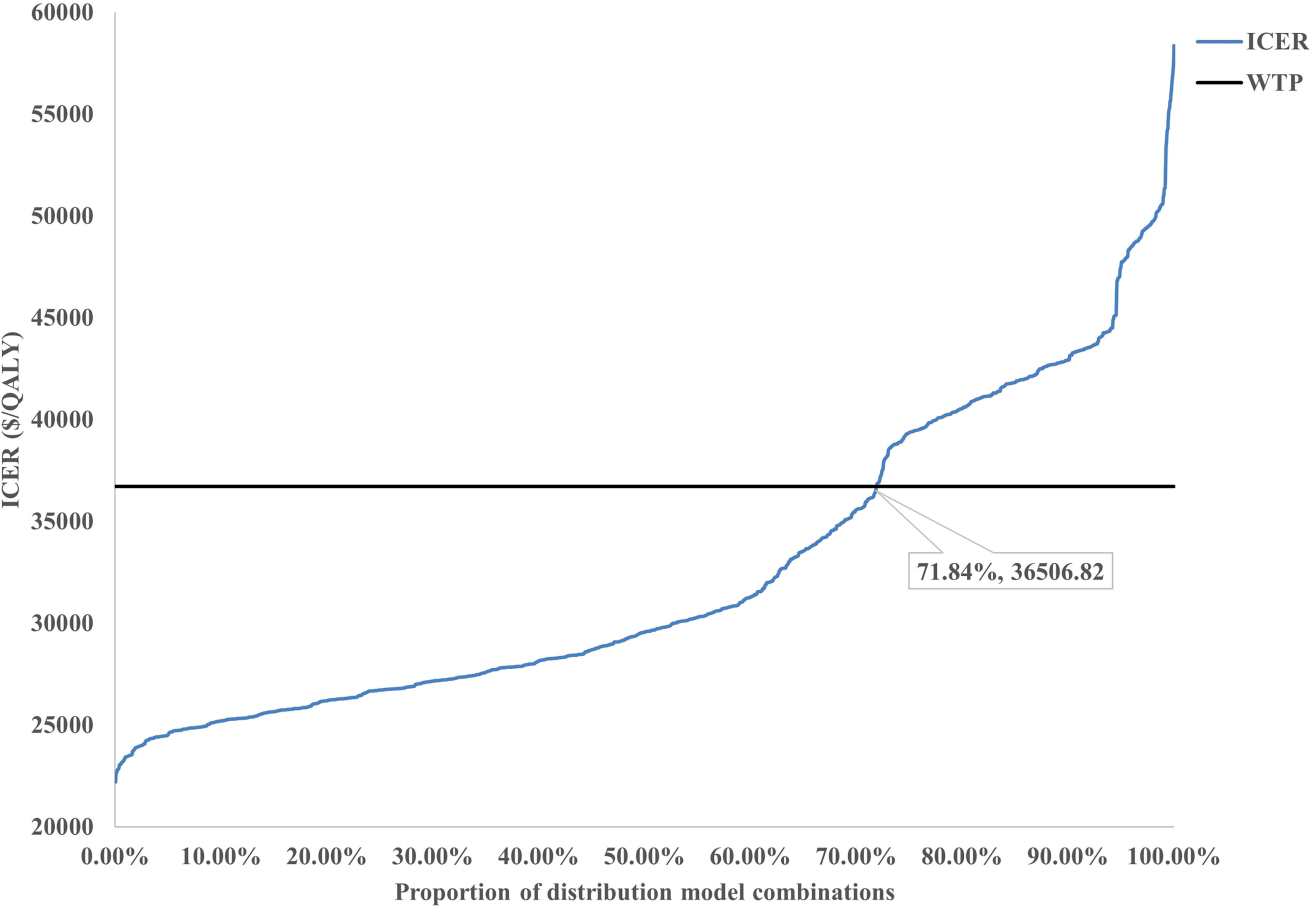
The line chart of the sensitivity analysis for the distribution model.

Scenario Analysis 2: To verify the robustness of the distribution model combinations, a systematic cross-validation process was designed in this study. Through screening, 72 valid control group distribution model combinations and 34 immunotherapy distribution group model combinations were identified, resulting in a total of 2,448 model pairing schemes for cross-validation. During the validation process, an algorithm was employed to evaluate the survival curves generated by each model combination, ensuring that the OS curve was consistently greater than or equal to the PFS curve. Based on the screened effective model combinations, transition probability matrices corresponding to each set of PFS and OS curves were calculated to describe the dynamic transitions of patients between different health states. The results demonstrated that the ICERs of all valid model combinations ranged from $22,204/QALY to $58,360/QALY, with the specific distribution shown in **Figure 6**. Among all valid combinations, 71.84% supported the cost-effectiveness of the immunotherapy group, with ICERs below the WTP threshold of $36,721.86/QALY established for China. This indicates that the treatment group exhibited significant economic advantages in the majority of model combinations. This indicates that the treatment group exhibited significant economic advantages in the majority of model combinations. The relevant data distribution is presented in **Figure 7**.

## 4 Discussion

This study evaluated the cost-effectiveness of ACI combined with chemotherapy versus chemotherapy alone in Chinese patients with mCRC by constructing a Markov model. The results demonstrated that ACI significantly prolongs patient survival and improves quality of life. However, the ACI group yielded an ICER of $35,881.71/QALY, which was marginally below China’s WTP threshold of $36,721.86/QALY (3× GDP). This analysis was based on a base-case ACI price of $6,819.45/cycle. Furthermore, price analysis indicated that when the ICER equals the WTP threshold at 1.5× GDP, or $18,360.93, the per-cycle cost of ACI needs to be reduced to $1,670.33. Similarly, when ICER equals WTP at 1.94× GDP ($23,746.80), the per-cycle cost must be reduced to $3,271.06 to achieve economic feasibility.

### 4.1 Justification of cost-effectiveness thresholds

In China, WTP threshold was set at $36,721.86/QALY (3× GDP). Two additional thresholds were incorporated into the price simulation analysis: $18,360.93/QALY (1.5× GDP) and $23,746.80/QALY (1.94× GDP), with justifications as follows:

The China Guidelines for Pharmacoeconomic Evaluations (2020) explicitly recommends using 1–3× GDP as the WTP threshold range, specifying that the upper bound (3× GDP) applies to interventions delivering significant survival benefits for life-threatening conditions (15). This aligns with global practices where higher multiples (e.g., 3× GDP) are adopted for oncology therapies with curative potential.

Based on Cai et al.’s statistical life value (VSL) framework (30), the monetary value of a QALY was estimated at 1.5× GDP for general health gains, increasing to 1.94× GDP for end-stage diseases (31). The extended threshold analysis at 1.94× GDP reflects disease severity adjustments, while the 3×GDP threshold captures societal prioritization of metastatic cancer treatment.

### 4.2 Limitations

This study had several limitations. Firstly, the model was based on data from the NCT03950154 clinical trial, which had a relatively small sample size, potentially limiting the generalizability of the results. Secondly, only direct medical costs were considered, while indirect costs (e.g., productivity loss) were excluded, possibly underestimating the total economic burden.

Cost Scope: Indirect costs (e.g., productivity loss) were excluded, potentially underestimating the economic burden.

Price Uncertainty: The base-case ACI price ($6,819.45/cycle) was extrapolated from assumptions derived from existing literature and expert consultations. Actual market pricing may vary, necessitating reassessment upon commercial availability.

Model Assumptions: Survival extrapolation beyond trial follow-up, though validated through 2,448 model combinations, may not fully capture long-term outcomes.

Finally, assumptions and parameter settings of the model may not fully reflect real-world complexities. Future studies should validate these findings through larger clinical trials or real-world data.

## 5 Conclusion

From the perspective of China’s healthcare system, ACI combined with chemotherapy has demonstrated significant clinical benefits for patients with mCRC, with the ICER approaching but remaining marginally below WTP threshold of three times GDP. Pricing analysis suggests that decision-makers can benefit from the pricing strategies outlined in this study to inform optimal decision-making. Further evidence is required to validate and refine the findings.

## Data Availability

The original contributions presented in the study are included in the article/**Supplementary Material**. Further inquiries can be directed to the corresponding authors.
