# Supplementary material for: Adoptive cellular immunotherapy combined with chemotherapy versus chemotherapy alone in Chinese patients with metastatic colorectal cancer: a cost-effectiveness analysis to inform drug pricing
Source: Front Oncol. 2025 Jun 9;15:1590319. doi: 10.3389/fonc.2025.1590319 (PMC12150851; doi:10.3389/fonc.2025.1590319)
Supplement: Supplementary file 1 [file DataSheet1.docx]

**Adoptive Cellular Immunotherapy Combined with Chemotherapy versus Chemotherapy Alone in Chinese Patients with Metastatic Colorectal Cancer: A Cost-Effectiveness Analysis to Inform Drug Pricing**

Liman Huo^1†^, Ping Liang^1†^, Yangyang Duan ^3^, Yanmei Xu^4^, Jianhua Tang ^2,5^, Qi Lv^2*^ and Rui Feng^2*^

1The Fourth Hospital of Hebei Medical University, Shijiazhuang 050011, China

2 School of Disaster and Emergency Medicine, Tianjin University, Tianjin 300072, PR China

3 School of Pharmacy, Hebei Medical University, Shijiazhuang 050017, China

4 Hebei Institute for Drug and Medical Device Control, Shijiazhuang 050011, China

5 The First Affiliated Hospital of Hebei North University, Zhangjiakou, Hebei, China

^*^Qi Lv and Rui Feng contributed equally to this work and are co-corresponding authors.

^*^Correspondence: Rui Feng, Email: [48100995@hebmu.edu.cn](mailto:48100995@hebmu.edu.cn)

^*^ Correspondence: Qi Lv, Email: [lvqi@tju.edu.cn](mailto:lvqi@tju.edu.cn)

^†^These authors contributed equally to this work and shared first authorship.

**Supplementary Material**

**Table S1 Baseline characteristics of patients in NCT03950154**

| Characteristic | All patients (n=202) | Immunotherapy  group (n=100) | Control group (n=102) |
| --- | --- | --- | --- |
| Sex | | | |
| Male | 104 (51.5%) | 45 (45.0%) | 59 (57.8%) |
| Female | 98 (48.5%) | 55 (55.0%) | 43(42.2%) |
| Age | | | |
| <60 years | 111 (55.0%) | 59 (59.0%) | 52 (51.0%) |
| ≥60 years | 91 (45.0%) | 41 (41.0%) | 50 (49.0%) |
| ECOG performance status | | | |
| 0 | 6 (3.0%) | 3 (3.0%) | 3 (2.9%) |
| 1 | 192 (95.0%) | 94 (94.0%) | 98 (96.1%) |
| 2 | 4 (2.0%) | 3 (3.0%) | 1 (1.0%) |
| Primary tumor site | | | |
| Left-sided | 122 (60.4%) | 63 (63.0%) | 59 (57.8%) |
| Right-sided | 80 (39.6%) | 37 (37.0%) | 43 (42.2%) |
| Metastatic sites | | | |
| Liver | 136 (67.3%) | 66 (66.0%) | 70 (68.6%) |
| Liver not affected | 66 (32.7%) | 34 (34.0%) | 32 (31.4%) |
| Number of metastatic sites | | | |
| 1 | 90 (44.6%) | 47 (47.0%) | 43 (42.2%) |
| ≥2 | 112 (55.4%) | 53 (53.0%) | 59 (57.8%) |
| Previous adjuvant chemotherapy | | | |
| No | 177 (87.6%) | 86 (86.0%) | 91 (89.2%) |
| Yes | 25 (12.4%) | 14 (14.0%) | 11 (10.8%) |
| RAS status | | | |
| Wild-type | 98 (48.5%) | 51 (51.0%) | 47 (46.1%) |
| Mutant | 76 (37.6%) | 35 (35.0%) | 41 (40.2%) |
| Missing data | 28 (13.9%) | 14 (14.0%) | 14 (13.7%) |
| BRAF status | | | |
| Wild-type | 157 (77.7%) | 79 (79.0%) | 78 (76.5%) |
| Mutant | 14 (6.9%) | 6 (6.0%) | 8 (7.8%) |
| Missing data | 31 (15.3%) | 15 (15.0%) | 16 (15.7%) |
| Mismatch repair status | | | |
| Proficient | 173 (85.6%) | 85 (85.0%) | 88 (76.3%) |
| Deficient | 5 (2.5%) | 3 (3.0%) | 2 (2.0%) |
| Missing data | 24 (11.9%) | 12 (12.0%) | 12 (11.8%) |

Control group, XELOX plus bevacizumab; Immunotherapy group, XELOX plus bevacizumab and PD-1 blocked-activated DC-CIK cells; ECOG: Eastern Cooperative Oncology Group; Data are presented as n (%).

**Table S2 Goodness-of-fit, extrapolation performance, and clinical rationality results of parametric survival models for 20-year model extrapolation.**

|  | OS | | | | PFS | | | |
| --- | --- | --- | --- | --- | --- | --- | --- | --- |
|  | Control group | | Immunotherapy group | | Control group | | Immunotherapy  group | |
| Model | AIC | BIC | AIC | BIC | AIC | BIC | AIC | BIC |
| Exp | 108.03 | 110.64 | **74.43** | **77.03** | 126.23 | 128.84 | 122.29 | 124.90 |
| Weibull | 101.33 | 106.54 | 76.12 | 81.33 | 106.03 | 111.24 | 113.01 | 118.22 |
| Gamma | 99.96 | 105.17 | 76.01 | 81.22 | 102.20 | 107.41 | 111.29 | 116.50 |
| lnorm | **97.82** | **103.03** | **75.31** | **80.52** | 100.28 | 105.49 | **110.38** | **115.59** |
| Gompertz | 106.46 | 111.67 | 76.39 | 81.60 | 117.63 | 122.84 | 118.96 | 124.18 |
| llogis | 99.13 | 104.34 | 75.48 | 80.69 | **99.68** | **104.89** | **110.33** | **115.54** |
| Gengamma | 99.59 | 107.41 | 77.33 | 85.14 | 102.28 | 110.10 | 112.02 | 119.84 |
| FP1 | 97.71 | 105.53 | 74.68 | 82.50 | 100.11 | 107.93 | 110.27 | 118.09 |
| FP2 | 99.00 | 109.42 | 71.37 | 81.79 | 101.14 | 111.56 | 112.05 | 122.47 |
| RCS | 101.22 | 109.03 | 71.78 | 84.81 | 104.57 | 114.99 | 114.79 | 125.21 |
| RP-hazard | 100.21 | 108.02 | 73.97 | 87.00 | 103.42 | 113.84 | 111.91 | 119.72 |
| RP-odds | 99.13 | 104.34 | 73.86 | 86.89 | **99.68** | **104.89** | **110.33** | **115.54** |
| RP-normal | **97.82** | **103.03** | 73.50 | 86.53 | 100.28 | 105.49 | 110.38 | 115.59 |
| GAM | 101.76 | 109.94 | 71.26 | 82.65 | 104.23 | 113.74 | 114.51 | 124.18 |
| Mix-cure | 151.34 | 159.15 | 120.72 | 136.35 | 122.23 | 130.05 | 154.73 | 162.54 |

OS, overall survival; PFS, progression-free survival; AIC, Akaike information criterion; BIC, Bayesian Information Criterion; Exp, exponential; lnorm, log normal; llogis, log logistic; gengamma, generalized gamma; FP, fractional polynomial; RCS, restricted cubic spline models; RP, Royston-Parmar models; GAM, Generalized Gamma Distribution. Bold and red data means that these AIC and BIC values are one of the smallest top three in this set of data. Bold red and highlighted data indicates the model that was ultimately selected for this study. Control group, XELOX plus bevacizumab; Immunotherapy group, XELOX plus bevacizumab and PD-1 blocked-activated DC-CIK cells.

**Table S3 Goodness-of-fit, extrapolation performance, and clinical rationality results of parametric survival models for 10-year model extrapolation.**

|  | OS | | | | PFS | | | |
| --- | --- | --- | --- | --- | --- | --- | --- | --- |
|  | Control group | | Immunotherapy group | | Control group | | Immunotherapy  group | |
| Model | AIC | BIC | AIC | BIC | AIC | BIC | AIC | BIC |
| Exp | 108.03 | 110.64 | **74.43** | **77.03** | 126.23 | 128.84 | 122.29 | 124.90 |
| Weibull | 101.33 | 106.54 | 76.12 | 81.33 | 106.03 | 111.24 | 113.01 | 118.22 |
| Gamma | **99.96** | **105.17** | 76.01 | 81.22 | 102.20 | 107.41 | 111.29 | 116.50 |
| lnorm | 97.82 | 103.03 | **75.31** | **80.52** | **100.28** | **105.49** | **110.38** | **115.59** |
| Gompertz | 106.46 | 111.67 | 76.39 | 81.60 | 117.63 | 122.84 | 118.96 | 124.18 |
| llogis | **99.13** | **104.34** | **75.48** | **80.69** | **99.68** | **104.89** | **110.33** | **115.54** |
| Gengamma | 99.59 | 107.41 | 77.33 | 85.14 | 102.28 | 110.10 | 112.02 | 119.84 |
| FP1 | **97.71** | **105.53** | 74.68 | 82.50 | 100.11 | 107.93 | 110.27 | 118.09 |
| FP2 | 99.00 | 109.42 | 71.37 | 81.79 | 101.14 | 111.56 | 112.05 | 122.47 |
| RCS | 101.22 | 109.03 | 71.78 | 84.81 | 104.57 | 114.99 | 114.79 | 125.21 |
| RP-hazard | 100.21 | 108.02 | 73.97 | 87.00 | 103.42 | 113.84 | 111.91 | 119.72 |
| RP-odds | 99.13 | 104.34 | 73.86 | 86.89 | **99.68** | **104.89** | **110.33** | **115.54** |
| RP-normal | 97.82 | 103.03 | 73.50 | 86.53 | **100.28** | **105.49** | **110.38** | **115.59** |
| GAM | 101.76 | 109.94 | 71.26 | 82.65 | 104.23 | 113.74 | 114.51 | 124.18 |
| Mix-cure | 151.34 | 159.15 | 120.72 | 136.35 | 122.23 | 130.05 | 154.73 | 162.54 |

OS, overall survival; PFS, progression-free survival; AIC, Akaike information criterion; BIC, Bayesian Information Criterion; Exp, exponential; lnorm, log normal; llogis, log logistic; gengamma, generalized gamma; FP, fractional polynomial; RCS, restricted cubic spline models; RP, Royston-Parmar models; GAM, Generalized Gamma Distribution. Bold and red data means that these AIC and BIC values are one of the smallest top three in this set of data. Bold red and highlighted data indicates the model that was ultimately selected for this study. Control group, XELOX plus bevacizumab; Immunotherapy group, XELOX plus bevacizumab and PD-1 blocked-activated DC-CIK cells.

**Table S4 Goodness-of-fit, extrapolation performance, and clinical rationality results of parametric survival models for 15-year model extrapolation.**

|  | OS | | | | PFS | | | |
| --- | --- | --- | --- | --- | --- | --- | --- | --- |
|  | Control group | | Immunotherapy group | | Control group | | Immunotherapy  group | |
| Model | AIC | BIC | AIC | BIC | AIC | BIC | AIC | BIC |
| Exp | 108.03 | 110.64 | **74.43** | **77.03** | 126.23 | 128.84 | 122.29 | 124.90 |
| Weibull | 101.33 | 106.54 | 76.12 | 81.33 | 106.03 | 111.24 | 113.01 | 118.22 |
| Gamma | 99.96 | 105.17 | 76.01 | 81.22 | 102.20 | 107.41 | 111.29 | 116.50 |
| lnorm | **97.82** | **103.03** | **75.31** | **80.52** | **100.28** | **105.49** | **110.38** | **115.59** |
| Gompertz | 106.46 | 111.67 | 76.39 | 81.60 | 117.63 | 122.84 | 118.96 | 124.18 |
| llogis | **99.13** | **104.34** | **75.48** | **80.69** | **99.68** | **104.89** | **110.33** | **115.54** |
| Gengamma | 99.59 | 107.41 | 77.33 | 85.14 | 102.28 | 110.10 | 112.02 | 119.84 |
| FP1 | 97.71 | 105.53 | 74.68 | 82.50 | 100.11 | 107.93 | 110.27 | 118.09 |
| FP2 | 99.00 | 109.42 | 71.37 | 81.79 | 101.14 | 111.56 | 112.05 | 122.47 |
| RCS | 101.22 | 109.03 | 71.78 | 84.81 | 104.57 | 114.99 | 114.79 | 125.21 |
| RP-hazard | 100.21 | 108.02 | 73.97 | 87.00 | 103.42 | 113.84 | 111.91 | 119.72 |
| RP-odds | **99.13** | **104.34** | 73.86 | 86.89 | **99.68** | **104.89** | **110.33** | **115.54** |
| RP-normal | **97.82** | **103.03** | 73.50 | 86.53 | **100.28** | **105.49** | **110.38** | **115.59** |
| GAM | 101.76 | 109.94 | 71.26 | 82.65 | 104.23 | 113.74 | 114.51 | 124.18 |
| Mix-cure | 151.34 | 159.15 | 120.72 | 136.35 | 122.23 | 130.05 | 154.73 | 162.54 |

Control group, XELOX plus bevacizumab; Immunotherapy group, XELOX plus bevacizumab and ACI.

**Table S5 Baseline values, ranges, and distributions of model parameters**

| Variable | Baseline value | Low | High | Distribution | Source |
| --- | --- | --- | --- | --- | --- |
| Costs of drugs ($/cycle) |  | | | | |
| Immunotherapy | X | Xmind | X_max_ | gamma |  |
| Oxaliplatin | 190.238 | 142.679 | 237.798 | gamma | ^[[1]](#endnote-1)^ |
| Capecitabine | 44.350 | 33.263 | 55.438 | gamma |  |
| Bevacizumab | 1293.730 | 970.297 | 1617.162 | gamma |  |
| FOLFIRI+Bevacizumab | 982.315 | 736.737 | 1227.894 | gamma | ^[[2]](#endnote-2)^ |
| FOLFIRI+Cetuximab | 1765.116 | 1323.837 | 2206.396 | gamma |  |
| FOLFIRI | 119.829 | 89.872 | 149.786 | gamma |  |
| Irinotecan+Capecitabine+Bevacizumab | 908.080 | 681.060 | 1135.100 | gamma |  |
| Irinotecan+Bevacizumab | 882.111 | 661.583 | 1102.639 | gamma |  |
| XELOX+Bevacizumab | 1078.972 | 809.229 | 1348.715 | gamma |  |
| Capecitabine+Bevacizumab | 888.456 | 666.342 | 1110.570 | gamma |  |
| Fruquintinib | 1034.639 | 775.979 | 1293.299 | gamma |  |
| Costs of administration ($/cycle) |  | | | | |
| Best supportive treatment | 293.773 | 220.329 | 367.216 | gamma | ^[[3]](#endnote-3)^,^[[4]](#endnote-4)^,^[[5]](#endnote-5)^,^[[6]](#endnote-6)^ |
| Laboratory testing | 1531.838 | 1148.879 | 1914.798 | gamma |  |
| Imaging examination | 42.306 | 31.729 | 52.882 | gamma |  |
| End of life care | 4469.477 | 3352.107 | 5586.846 | gamma |  |
| Costs of serious TRAEs ($/cycle) |  | | | | |
| Anemia | 1089.505 | 817.129 | 1361.882 | gamma | 2,3,4,5 |
| Leukopenia | 2309.392 | 1732.044 | 2886.739 | gamma |  |
| Thrombocytopenia | 3809.853 | 2857.390 | 4762.317 | gamma |  |
| Hand-foot syndrome | 2042.336 | 1531.752 | 2552.920 | gamma |  |
| Diarrhea | 1005.976 | 754.482 | 1257.471 | gamma |  |
| Oral mucositis | 1481.468 | 1111.101 | 1851.835 | gamma |  |
| Disutility of serious TRAEs |  | | | | |
| Utility of anemia | 0.085 | 0.064 | 0.106 | beta | ^[[7]](#endnote-7)^,^[[8]](#endnote-8)^,^[[9]](#endnote-9)^,^[[10]](#endnote-10)^,^[[11]](#endnote-11)^,^[[12]](#endnote-12)^ |
| Utility of leukopenia | 0.090 | 0.068 | 0.113 | beta |  |
| Utility of thrombocytopenia | 0.037 | 0.0259 | 0.0481 | beta |  |
| Utility of hand-foot syndrome | 0.108 | 0.012 | 0.020 | beta |  |
| Utility of oral mucositis | 0.269 | 0.202 | 0.336 | beta |  |
| Utility of diarrhea | 0.047 | 0.035 | 0.059 | beta |  |
| PB-Utility |  | | | | |
| Utility of progression-free survival in test group | 0.8500 | 0.5950 | 1.1050 | beta | ^[[13]](#endnote-13)^,^[[14]](#endnote-14)^ |
| Utility of progression-free survival in control group | 0.8000 | 0.5600 | 1.0400 | beta |  |
| Utility of overall survival | 0.650 | 0.488 | 0.813 | beta |  |
| Discount rate | 0.050 | 0.000 | 0.080 | beta | ^[[15]](#endnote-15)^ |
| Discount rate_period_ | 0.003 | 0.000 | 0.005 | beta |  |
| Risk of TRAEs |  | | | | |
| Immunotherapy group |  | | | | |
| Leukopenia | 0.060 | 0.045 | 0.075 | beta | ^[[16]](#endnote-16)^ |
| Thrombocytopenia | 0.020 | 0.015 | 0.025 | beta |  |
| Anemia | 0.030 | 0.023 | 0.038 | beta |  |
| Hand-foot syndrome | 0.040 | 0.030 | 0.050 | beta |  |
| Diarrhea | 0.020 | 0.015 | 0.025 | beta |  |
| Oral mucositis | 0.010 | 0.008 | 0.013 | beta |  |
| Control group |  | | | | |
| Leukopenia | 0.049 | 0.037 | 0.061 | beta | 15 |
| Thrombocytopenia | 0.039 | 0.029 | 0.049 | beta |  |
| Anemia | 0.020 | 0.015 | 0.025 | beta |  |
| Hand-foot syndrome | 0.039 | 0.029 | 0.049 | beta |  |
| Diarrhea | 0.049 | 0.037 | 0.061 | beta |  |
| Oral mucositis | 0.020 | 0.015 | 0.025 | beta |  |
| Proportions of subsequent treatment |  | | | | |
| Immunotherapy group |  |  |  |  |  |
| FOLFIRI+Bevacizumab | 0.210 | 0.158 | 0.263 | beta | 15, ^2^ |
| FOLFIRI+Cetuximab | 0.030 | 0.023 | 0.038 | beta |  |
| FOLFIRI | 0.010 | 0.008 | 0.013 | beta |  |
| Irinotecan+Capecitabine+Bevacizumab | 0.010 | 0.008 | 0.013 | beta |  |
| Irinotecan+Bevacizumab | 0.020 | 0.015 | 0.025 | beta |  |
| XELOX+Bevacizumab | 0.010 | 0.008 | 0.013 | beta |  |
| Capecitabine+Bevacizumab | 0.030 | 0.023 | 0.038 | beta |  |
| Fruquintinib | 0.020 | 0.015 | 0.025 | beta |  |
| Best supportive treatment | 0.110 | 0.083 | 0.138 | beta | 15 |
| Control group |  |  |  |  | 15 |
| FOLFIRI+Bevacizumab | 0.284 | 0.213 | 0.355 | beta |  |
| FOLFIRI+Cetuximab | 0.020 | 0.015 | 0.025 | beta |  |
| FOLFIRI | 0.029 | 0.022 | 0.036 | beta |  |
| Irinotecan+Capecitabine+Bevacizumab | 0.029 | 0.022 | 0.036 | beta |  |
| Irinotecan+Bevacizumab | 0.020 | 0.015 | 0.025 | beta |  |
| XELOX+Bevacizumab | 0.020 | 0.015 | 0.025 | beta |  |
| Capecitabine+Bevacizumab | 0.020 | 0.015 | 0.025 | beta |  |
| Fruquintinib | 0.020 | 0.015 | 0.025 | beta |  |
| Best supportive treatment | 0.055 | 0.041 | 0.069 | beta | 15 |

Control group, XELOX plus bevacizumab; Immunotherapy group, XELOX plus bevacizumab and PD-1 blocked-activated DC-CIK cells.TRAEs, treatment-related adverse events; PB-utility, progression-based utility; FOLFIRI, Irinotecan plus Leucovorin plus 5-FU; XELOX, Oxaliplatin plus Capecitabine;Discount rate_period_, the 21-day discount rate.

**Table S6 Administration and dosage of second-line treatment regimens**

| Treatment | Dosage and administration | References |
| --- | --- | --- |
| FOLFIRI + Bevacizumab | Irinotecan: 180 mg/m², IV infusion, Day 1 Leucovorin: 400 mg/m², IV infusion, Day 1 5-FU: 400 mg/m² IV bolus + 2400 mg/m² continuous IV infusion over 46 hours Bevacizumab: 7.5 mg/kg（every 3 weeks）, IV infusion | 16 |
| FOLFIRI + Cetuximab | Irinotecan: 180 mg/m², IV infusion, Day 1 Leucovorin: 400 mg/m², IV infusion, Day 1 5-FU: 400 mg/m² IV bolus + 2400 mg/m² continuous IV infusion over 46 hours Cetuximab: Initial dose 400 mg/m², maintenance dose 250 mg/m², weekly, IV infusion |  |
| FOLFIRI | Irinotecan: 180 mg/m², IV infusion, Day 1 Leucovorin: 400 mg/m², IV infusion, Day 1 5-FU: 400 mg/m² IV bolus + 2400 mg/m² continuous IV infusion over 46 hours |  |
| Irinotecan + Capecitabine + Bevacizumab | Irinotecan: 250 mg/m², IV infusion，every 3 weeks Capecitabine: 1000 mg/m², oral, twice daily, for 14 days, every 21 days per cycle  Bevacizumab: 7.5 mg/kg, IV infusion, every 3 weeks |  |
| Irinotecan + Bevacizumab | Irinotecan: 180 mg/m², IV infusion, every 2 weeks Bevacizumab: 5 mg/kg, IV infusion, every 2 weeks |  |
| XELOX + Bevacizumab | Oxaliplatin: 130 mg/m², IV infusion, Day 1 Capecitabine: 1000 mg/m², oral, twice daily, for 14 days, every 21 days per cycle Bevacizumab: 7.5 mg/kg, IV infusion, every 3 weeks |  |
| Capecitabine + Bevacizumab | Capecitabine: 1000 mg/m², oral, twice daily, for 14 days, every 21 days per cycle Bevacizumab: 7.5 mg/kg, IV infusion, every 3 weeks |  |
| Fruquintinib | Fruquintinib: 5 mg, oral, once daily, for 21 days, every 28 days per cycle |  |


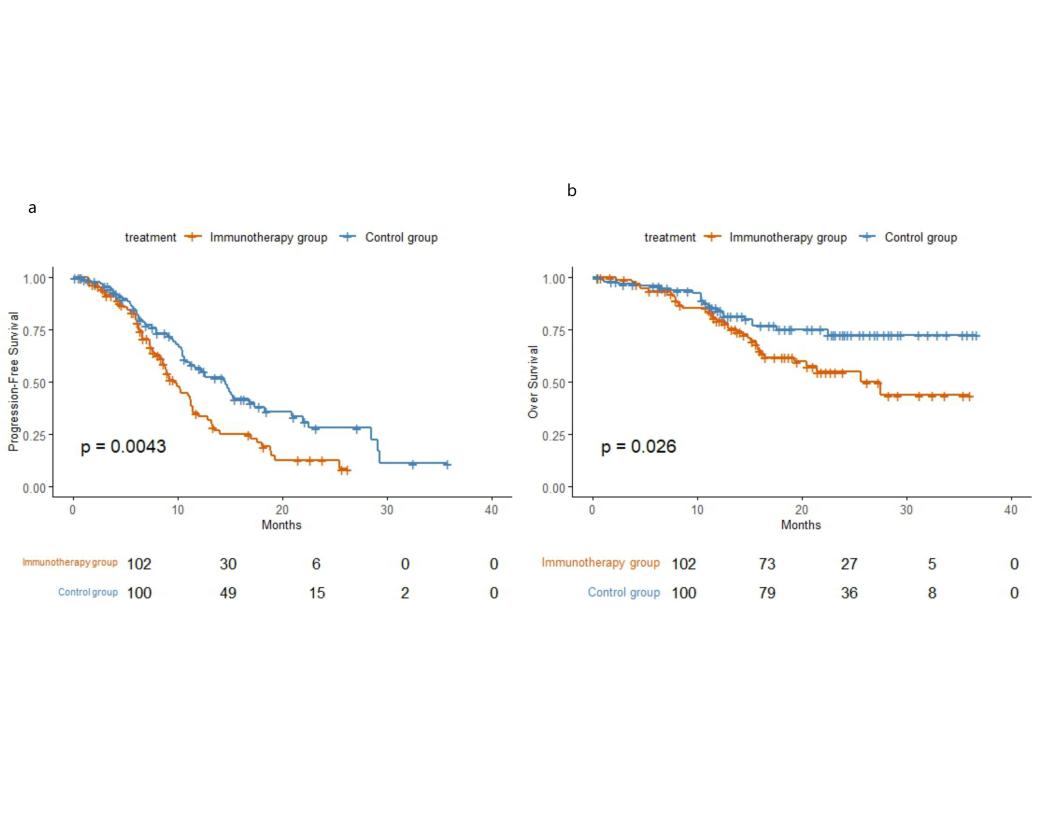


**Figure S1. K-M survival analysis in the in the intention-to-treat population.** (a) K-M estimates of PFS in the intention-to-treat population. (b) K-M estimates of OS in the intention-to-treat population. Crosses denote censored patients. Control group, XELOX plus bevacizumab; Immunotherapy group, XELOX plus bevacizumab and PD-1 blocked-activated DC-CIK cells.

**
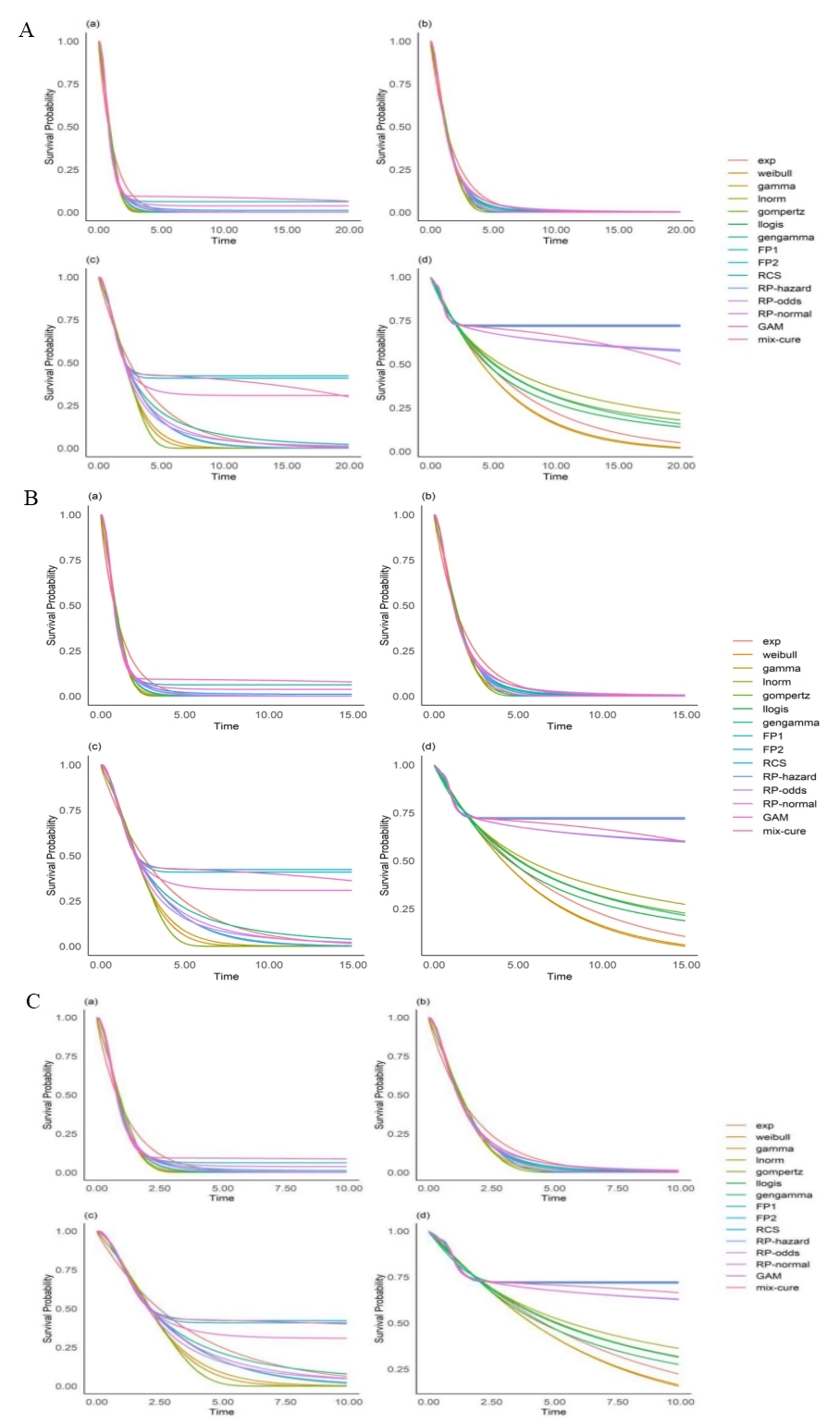
**

**Figure S2. Goodness-of-fit analysis of parametric survival models against (K-M) curves across varying simulation durations.** (A) 20-year, (B) 15-year, and (C) 10-year model simulations. Subpanels (a) PFS in the control group; (b) PFS in the immunotherapy group; (c) OS in the control group; (d) OS in the immunotherapy group. Control group, XELOX plus bevacizumab; Immunotherapy group, XELOX plus bevacizumab and PD-1 blocked-activated DC-CIK cells.

**
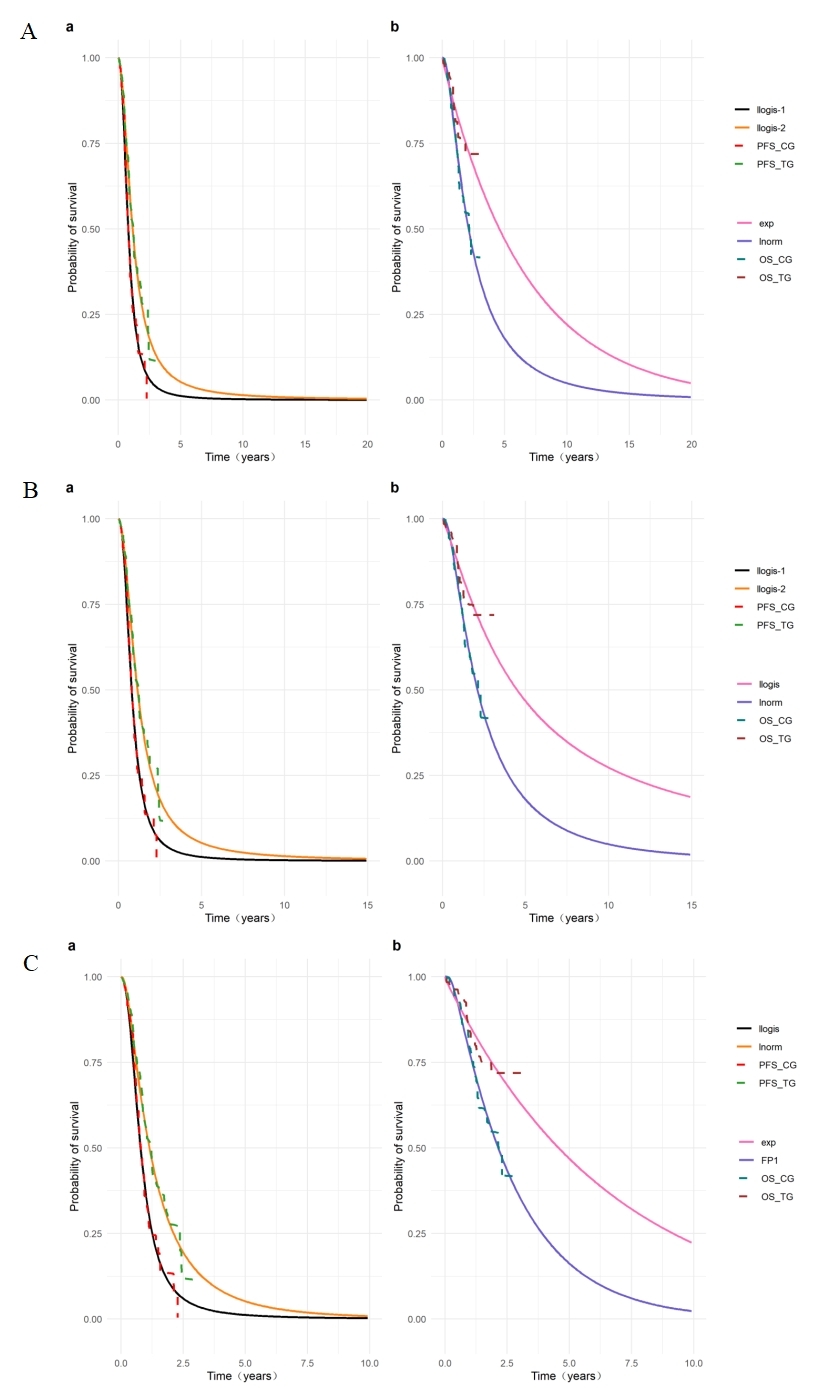
**

**Figure S3. Comparative goodness-of-fit analysis of parametric survival models and K-M curves across distinct simulation durations.** (A) 20-year, (B) 15-year and (C) 10-year model simulations. Subpanels: (a) PFS in TG and CG; (b) OS in TG and CG. Dashed lines depict the original K-M curves; solid lines represent parametric survival models with optimal distribution fits. llogist-1, log-logistic distribution model for PFS in the control group; llogist-2, log-logistic distribution model for PFS in the immunotherapy group; PFS_TG, PFS in the immunotherapy group; PFS_CG, PFS in the control group; OS_TG, OS in the immunotherapy group; OS_CG, OS in the control group. Control group, XELOX plus bevacizumab; Immunotherapy group, XELOX plus bevacizumab and PD-1 blocked-activated DC-CIK cells.

**Supplementary Validation Analysis**

To validate the rationale for model selection, we performed a systematic comparative analysis between the Markov model and the partitioned survival model (PSM). The results of this analysis are documented in the file "Validation analysis of partition survival model and Markov model.xlsx" on GitHub (https://github.com/Huoliman/HuoDate). Specifically, in the worksheets "TG state" and "CG state", the state distributions of both models across different time points demonstrate substantial alignment **(Figure S4)**, indicating minimal divergence between the Markov model and PSM in simulating patient survival states. Furthermore, a foundational comparative analysis based on the PSM was conducted (Table S7), revealing negligible differences in the ICERs calculated by both models, thereby corroborating their compatibility in cost-effectiveness analyses. The observed minor discrepancies can be attributed to two key factors: (1) Life-table method integration: The Markov model, implemented via the heemod package, incorporates the life-table method for calibration, enhancing simulation accuracy. In contrast, the PSM in the Excel framework lacks analogous calibration, potentially introducing subtle deviations. (2) Tunnel state precision: The Markov model enables precise estimation of post-progressive disease (PD) treatment costs through tunnel states, whereas the PSM in this study could not achieve equivalent granularity in modeling tunnel states, limiting its capacity for detailed cost calculations.

In conclusion, our analysis demonstrates consistent outcomes between the two models despite methodological differences. The Markov model, however, better fulfills the requirement for accurate cost estimation of post-PD treatments in this study, justifying its selection as the primary analytical tool. These findings provide critical insights for model selection in future studies with similar objectives.

**Table S7 Comparative Analysis of Markov Models and Partitioned Generative Models**

|  | Group | Total cost ($) | Total QALYs | Incremental cost ($/QALY) |
| --- | --- | --- | --- | --- |
| PSM | Immunotherapy group | 105651.2975 | 3.761063454 | 60939.81654 |
|  | Control group | 44711.48094 | 2.079583556 |  |
| Markov | Immunotherapy group | 108136.35 | 3.914831 | 62029.79 |
|  | Control group | 46106.57 | 2.186101 |  |

Control group, XELOX plus bevacizumab; Immunotherapy group; Immunotherapy group, XELOX plus bevacizumab and PD-1 blocked-activated DC-CIK cells.


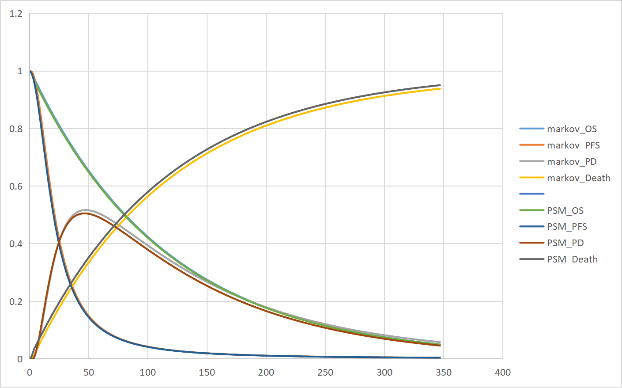

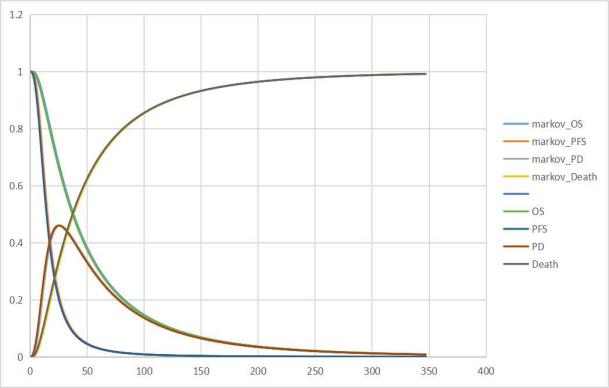


a

b

**Figure S4. Comparison of Partitioned Model Generation in Markov Models**

To ensure the selection of the most relevant and reliable utility values, we performed a comprehensive literature search across multiple databases, including CNKI (China National Knowledge Infrastructure), Wanfang Database, PubMed, and Embase. The specific search strategies employed for each database are detailed below:

PubMed Search Strategy: ("Cost-Effectiveness Analysis"[Mesh]) OR ((((((((Analysis, Cost-Effectiveness) OR (Cost Effectiveness Analysis)) OR (Cost Effectiveness)) OR (Effectiveness, Cost)) OR (Cost Effectiveness Ratio)) OR (Cost Effectiveness Ratios)) OR (Effectiveness Ratio, Cost)) OR (Ratio, Cost Effectiveness)) AND (y_5 [Filter])) AND (((((((((((((((Colorectal Neoplasm) OR (Neoplasm, Colorectal)) OR (Colorectal Tumors)) OR (Colorectal Tumor)) OR (Tumor, Colorectal)) OR (Tumors, Colorectal)) OR (Neoplasms, Colorectal)) OR (Colorectal Cancer)) OR (Cancer, Colorectal)) OR (Cancers, Colorectal)) OR (Colorectal Cancers)) OR (Colorectal Carcinoma)) OR (Carcinoma, Colorectal)) OR (Carcinomas, Colorectal)) OR (Colorectal Carcinomas))

Embase Search Strategy: ('cost effectiveness'/exp OR 'cost benefit analysis'/exp OR 'cost effectiveness analysis' OR 'cost effectiveness ratio' OR 'cost effectiveness ratios' OR 'effectiveness ratio cost' OR 'ratio cost effectiveness') AND ([embase]/lim NOT [medline]/lim) AND ('colorectal neoplasm'/exp OR 'colorectal tumor'/exp OR 'colorectal cancer'/exp OR 'colorectal carcinoma'/exp OR 'colorectal neoplasms' OR 'colorectal tumors' OR 'colorectal cancers' OR 'colorectal carcinomas')。

Using the above search strategies, we identified studies related to cost-effectiveness analyses of mCRC. From these studies, we systematically extracted utility value data, which are summarized in **Table S8 and Table S9**.

In conducting the systematic literature review, we identified that existing utility estimates might be contextually derived from different clinical scenarios or inadequately representative of health state preferences in our target mCRC population. To address these limitations, we established the following standardized selection criteria for utility value extraction:

Population specificity criterion: Prioritized utility data directly derived from mCRC patient cohorts; Health state differentiation criterion: Required explicit distinction of utility estimates between distinct disease progression phases; Measurement standardization criterion: Restricted inclusion to utilities obtained through internationally validated instruments (EQ-5D or SF-6D) to ensure cross-study comparability. Through this tripartite screening process, the utility parameters from Chongqing T etal. (2021) were selected as baseline values. For adverse event-related disutilities, we systematically identified and prioritized evidence specifically generated from mCRC populations, establishing the following disutility values: anemia (0.085), leukopenia (0.0607), thrombocytopenia (0.037), hand-foot syndrome (0.108), diarrhea (0.029), and oral mucositis (0.06). To evaluate the impact of utility value uncertainty on cost-effectiveness outcomes, we conducted sensitivity analyses employing a ±30% variation range. This threshold selection was informed by both the variability reported in existing literature and established parameters for sensitivity analyses in health economic evaluations.

This methodological framework enhances the robustness of our findings against potential utility value fluctuations while maintaining alignment with current health technology assessment standards. The rigorous selection process and comprehensive sensitivity testing collectively strengthen the validity of our economic model outputs.

**Table S8 Summary of Population-Based Utility Values Reported in the Literature**

| Parameter | Baseline | Low Bound | High Bound | References |
| --- | --- | --- | --- | --- |
| Utility of PFS (USA/UK) | 0.73 | 0.584 | 0.876 | ^13^^,^^[[17]](#endnote-17),^^[[18]](#endnote-18)^^,^^[[19]](#endnote-19)^ |
| Utility of PD (USA/UK) | 0.59 | 0.472 | 0.708 |  |
| Utility of PFS (China) | 0.78 | 0.624 | 0.936 |  |
| Utility of PD (CN) | 0.69 | 0.552 | 0.828 |  |
| Utility of PFS | 0.85 | 0.765 | 0.935 |  |
| Utility of PD | 0.68 | 0.612 | 0.748 |  |
| Utility of PFS in treatment group | 0.85 | 0.84 | 0.87 |  |
| Utility of PFS in control group | 0.80 | 0.80 | 0.78 |  |
| Utility of PD | 0.73 | 0.70 | 0.76 |  |
| Utility of PFS in treatment group | 0.84 | 0.67 | 1.00 |  |
| Utility of PFS in control group | 0.77 | 0.62 | 0.92 |  |
| Utility of PD | 0.65 | 0.52 | 0.78 | ^[[20]](#endnote-20),^^[[21]](#endnote-21)^^,^^[[22]](#endnote-22),^^[[23]](#endnote-23),^^[[24]](#endnote-24)^ |
| Utility of PFS | 0.84 | 0.62 | 0.92 |  |
| Utility of PD | 0.57 | 0.52 | 0.78 |  |
| Utility of PFS | 0.84 | 0.62 | 0.92 |  |
| Utility of PD | 0.57 | 0.52 | 0.78 |  |
| Utility of PFS | 0.82 | 0.62 | 0.92 |  |
| Utility of PD | 0.64 | 0.52 | 0.78 |  |
| Utility of PFS | 0.78 | - | - |  |
| Utility of PD | 0.66 | - | - |  |

**Table S9 Utility Decrements of Treatment-Related Adverse Events Reported in Literature**

| Parameter | Baseline | References |
| --- | --- | --- |
| Utility of anemia | 0.085 | ^1,6,7,8,9,23^ |
| Utility of anemia | 0.024 |  |
| Utility of thrombocytopenia | 0.037 |  |
| Utility of anemia | 0.085 |  |
| Utility of diarrhea | 0.090 |  |
| Utility of leukopenia | 0.0607 |  |
| Utility of hand-foot syndrome | 0.108 |  |
| Utility of diarrhea | 0.029 |  |
| Utility of thrombocytopenia | 0.025 |  |
| Utility of oral mucositis | 0.06 |  |

**Sensitivity Analysis and ICER Formula Derivation**

Key Definitions , Control Group: Patients received XELOX plus bevacizumab (standard chemotherapy regimen); Treatment Group: Patients received XELOX plus bevacizumab combined with PD-1 blocked-activated DC-CIK cells (immunotherapy regimen). Mathematical Formulation:

$ICER=\frac{\Delta Cost}{\Delta QALY}$ ,where $\Delta Cost={Cost}_{Treatment}-{Cost}_{Control}$*, and* $\Delta QALY={QALY}_{Treatment}-{QALY}_{Control}$

The ICER formula under the study’s assumptions is expressed as:

$ICER=\frac{C_{imu,treatment}+(C_{other,treatment}-C_{control})}{\Delta QALY}$*,*

Where:

$C_{imu,treatment}:Cost of immunotherapy regimen$ *C_imu.*

$C_{other,treatment}$*:Non-immunotherapy costs in the treatment group.*

$C_{control}$*:Total costs in the control group.*

$$\Delta QALY={QALY}_{treatment}-{QALY}_{control}>0.$$

Simplified Relationship , in the OWSA, all parameters except $C_{imu,treatment}$ were fixed.

The ICER simplifies to:

$$ICER=\underset{Constant(a)}{\underbrace{\frac{1}{\Delta QALY}}}\cdot C_{imu,treatment}+\underset{Constant(b)}{\underbrace{\frac{C_{other,treatment}-C_{control}}{\Delta QALY}}}$$

This relationship $ICER=a\cdot C_{imu}+b$ explains the positive correlation between C_imu price and ICER observed in Figure 5.

Assumptions and Validation:

1. Fixed parameters: $\Delta QALY, C_{other,treatment}$ and $C_{control}$ were held constant. Clinical efficacy (QALY gains) and non-immunotherapy costs were invariant to C_imu price changes.
2. Validation of positive: The proportionality constant $a=\frac{1}{\Delta QALY}$ was derived from trial data $(\Delta QALY=1.73)$ . The ICER values scale positive with $C_{imu}$, consistent with the theoretical model.

**Discount Rate Conversion Methodology**

The annual discount rate (5%) reported in the manuscript adheres to standard health economic practices. For model calculations, we converted this annual rate to a 21-day periodic discount rate, as detailed in Table S5 (Supplementary Materials).

Formula for Periodic Discount Rate:

Let $r_{annual}=5\%$ denote the annual discount rate, and$T=21$days represent the cycle length (assuming 365 days per year). The periodic discount rate $r_{period}$ is calculated as:

$$(1+r_{period})^{365/T}=1+r_{annual}$$

Solve the equation to get:

$r_{period}=(1+r_{annual})^{T/365}-1$

Parameter substitution and calculation Substitute$(r_{annual}=0.05) and (T=21)$into the formula:

$$r_{period}=(1+0.05)^{21/365}-1\approx0.00273$$

Consistency was validated by reverse-calculation:

$$(1+r_{period})^{365/21}-1\approx0.05$$

**Code availability**

The models and codes for reproducing this study can be found on GitHub (https://github.com/Huoliman/HuoDate).

**References**

1. Wuxuwang. (n.d.) Available at: https://www.wuxuwang.com/ [Accessed November 25, 2024]. [↑](#endnote-ref-1)
2. Chen L, Hu H, Yuan Y, Weng S. CSCO guidelines for colorectal cancer version 2024: updates and discussions. Chin J Cancer Res. 2024;36(3):233–239.

   doi:10.21147/j.issn.1000-9604.2024.03.01 [↑](#endnote-ref-2)
3. Rui M, Fei Z, Wang Y, Xu X, Wu B. Cost-effectiveness analysis of sintilimab plus chemotherapy versus camrelizumab plus chemotherapy for the treatment of first-line locally advanced or metastatic nonsquamous NSCLC in China. J Med Econ. 2022;25(1):618–629. doi:10.1080/13696998.2022.2071066 [↑](#endnote-ref-3)
4. Bullement A, Nathan P, Willis A, Wong H, Paulden M. Cost effectiveness of avelumab for metastatic Merkel cell carcinoma. Pharmacoecon Open. 2019;3(3):377–390. doi:10.1007/s41669-018-0115-y [↑](#endnote-ref-4)
5. Wang K, Li S, Dou L, Sun Y, Liu Z. Cost-effectiveness analysis of pembrolizumab as first-line therapy for unresectable or metastatic MSI-H/dMMR colorectal cancer. Chin J New Drugs Clin Rem. 2024;43(3):229–235. doi:10.14109/j.cnki.xyylc.2024.03.13 [↑](#endnote-ref-5)
6. National Institute for Health and Care Excellence. Pembrolizumab for untreated metastatic colorectal cancer with high microsatellite instability or mismatch repair deficiency. (2021-06-21) [cited 2024-11-06]. Available from: https://www.nice.org.uk/guidance/ta709 [↑](#endnote-ref-6)
7. Goldstein DA, Ahmad BB, Chen Q, Ayer T, Howard DH. Cost-effectiveness analysis of regorafenib for metastatic colorectal cancer. J Clin Oncol. 2015;33(32):3727-32. doi:10.1200/JCO.2015.61.9569 [↑](#endnote-ref-7)
8. Lloyd A, Nafees B, Narewska J, Dewilde S, Watkins J. Health state utilities for metastatic breast cancer. Br J Cancer. 2006;95(6):683-690. doi: 10.1038/sj.bjc.6603326 [↑](#endnote-ref-8)
9. Al-Rudayni AHM, Gopinath D, Maharajan MK, Menon RK. Impact of oral mucositis on quality of life in patients undergoing oncological treatment: a systematic review. Transl Cancer Res. 2020;9(4):3126-3134. doi:10.21037/tcr.2020.02.77 [↑](#endnote-ref-9)
10. Su D, Wu B, Shi L. Cost-effectiveness of atezolizumab plus bevacizumab vs sorafenib as first-line treatment of unresectable hepatocellular carcinoma. JAMA Netw Open. 2021;4(2): e210037. doi:10.1001/jamanetworkopen.2021.0037 [↑](#endnote-ref-10)
11. MedValley. Taiwan approves first clinical hospital for autologous immune cell therapy, releases treatment price. (2023) [cited 2023 Oct 24]. Available from: https://m.medvalley.cn/article/223-0-58663 [↑](#endnote-ref-11)
12. Cai D, Shi S, Jiang S, Li H, Ding Z. Estimation of the cost-effective threshold of a quality-adjusted life year in China based on the value of statistical life. Eur J Health Econ. 2022; 23:607-615. doi:10.1007/s10198-021-01384-z [↑](#endnote-ref-12)
13. Tikhonova IA, Huxley N, Snowsill T, Crathorne L, Varley-Campbell J, Napier M, et al. Economic analysis of first-line treatment with cetuximab or panitumumab for RAS wild-type metastatic colorectal cancer in England. Pharmacoeconomics. 2018;36:837–851. doi:10.1007/s40273-018-0630-9 [↑](#endnote-ref-13)
14. Freeman K, Connock M, Cummins E, Gurung T, Taylor-Phillips S, Court R, etal. Fluorouracil plasma monitoring: systematic review and economic evaluation of the My5-FU assay for guiding dose adjustment in patients receiving fluorouracil chemotherapy by continuous infusion. Health Technol Assess. 2015;19(91):1-321. doi:10.3310/hta19910 [↑](#endnote-ref-14)
15. Chinese Pharmaceutical Association. China Guidelines for Pharmacoeconomic Evaluations (2020). (2020-11-28) [cited 2025 Apr 19]. Available from: https://www.cpa.org.cn/ [↑](#endnote-ref-15)
16. Pan QZ, Zhao JJ, Liu L, Zhang DS, Wang LP, Hu WW, etal. XELOX (capecitabine plus oxaliplatin) plus bevacizumab (anti-VEGF-A antibody) with or without adoptive cell immunotherapy in the treatment of patients with previously untreated metastatic colorectal cancer: a multicenter, open-label, randomized, controlled, phase 3 trial. Signal Transduct Target Ther. 2024;9(1):79. doi:10.1038/s41392-024-01788-2 [↑](#endnote-ref-16)
17. Tabernero J, Prager GW, Fakih M, Ciardiello F, Van Cutsem E, Elez E, etal. Trifluridine/tipiracil plus bevacizumab for third-line treatment of refractory metastatic colorectal cancer: The phase 3 randomized SUNLIGHT study. J Clin Oncol. 2023;41(4_suppl):4-10. doi:10.1200/JCO.2023.41.4_suppl.4 [↑](#endnote-ref-17)
18. Husereau D, Drummond M, Augustovski F, de Bekker-Grob E, Briggs AH, Carswell C, et al. Consolidated Health Economic Evaluation Reporting Standards 2022 (CHEERS 2022) statement: updated reporting guidance for health economic evaluations. J Health Econ Outcomes Res. 2022;10(1):1–11. doi:10.1177/23814683211061097 [↑](#endnote-ref-18)
19. Chongqing T, Sini L, Xiaohui Z, Liubao P, Ye P, Shuxia Q, etal. Cost-Effectiveness of First-Line Versus Second-Line Pembrolizumab or Chemotherapy in Patients With Microsatellite-Instability-High/Mismatch Repair-Deficient Advanced Colorectal Cancer. Front Pharmacol. 2021;12:802942. doi: 10.3389/fphar.2021.802942. [↑](#endnote-ref-19)
20. Li J, Qin S, Xu R, Yau TC, Ma B, Pan H, etal. Regorafenib plus best supportive

    care versus placebo plus best supportive care in Asian patients with previously treated

    metastatic colorectal cancer (CONCUR): a randomized, double-blind, placebo-controlled, phase 3 trial. Lancet Oncol. 2015; 16(6):619–629. doi: 10.1016/S1470-2045 (15)70156-7 [↑](#endnote-ref-20)
21. Ladabaum U, Mannalithara A, Meester RGS, Gupta S, Schoen RE. Cost-Effectiveness and National Effects of Initiating Colorectal Cancer Screening for Average-Risk Persons at Age 45 Years Instead of 50 Years. Gastroenterology. 2019 ;157(1):137-148.

    doi: 10.1053/j.gastro.2019.03.023. [↑](#endnote-ref-21)
22. Färkkilä N, Sintonen H, Saarto T, Järvinen H, Hänninen J, Taari K, etal. Health-related quality of life in colorectal cancer. Colorectal Dis. 2013;15(4):428–435. doi:10.1111/codi.12143 [↑](#endnote-ref-22)
23. Franken MD, de Hond A, Degeling K, Punt CJA, Koopman M, Uyl-de Groot CA, etal. Evaluation of the performance of algorithms mapping EORTC QLQ-C30 onto the EQ-5D index in a metastatic colorectal cancer cost-effectiveness model. Health Qual Life Outcomes. 2020;18(1):240-246. doi: 10.1186/s12955-020-01481-2. [↑](#endnote-ref-23)
24. Li J, Qin S, Xu R, Yau TC, Ma B, Pan H, etal. Regorafenib plus best supportive

    care versus placebo plus best supportive care in Asian patients with previously treated

    metastatic colorectal cancer (CONCUR): a randomized, double-blind, placebo-controlled, phase 3 trial. Lancet Oncol. 2015; 16(6):619–629. doi: 10.1016/S1470-2045 (15)70156-7 [↑](#endnote-ref-24)
